# Supplementary figures and images for: The development of autonomous unmanned aircraft systems for mosquito control
Source: PLoS One. 2020 Sep 18;15(9):e0235548. doi: 10.1371/journal.pone.0235548 (PMC7500627; doi:10.1371/journal.pone.0235548)

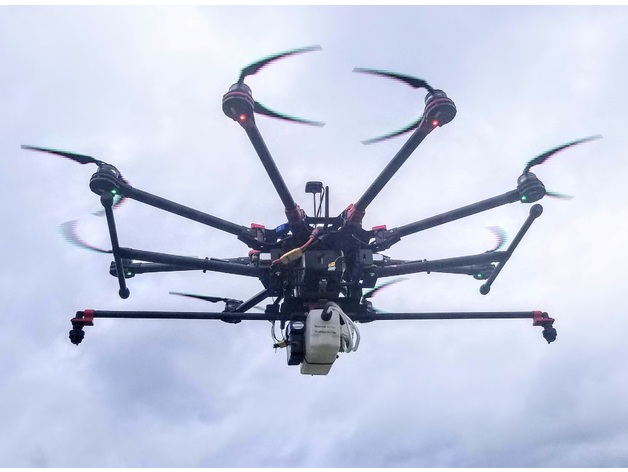

Supplement: S1 File — (ZIP) [file pone.0235548.s001.zip › images/20191017_111246_1.jpg]

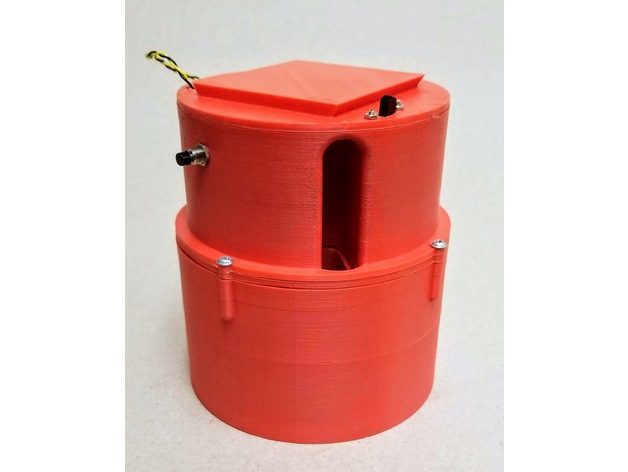

Supplement: S2 File — (ZIP) [file pone.0235548.s002.zip › images/20191008_141221.jpg]

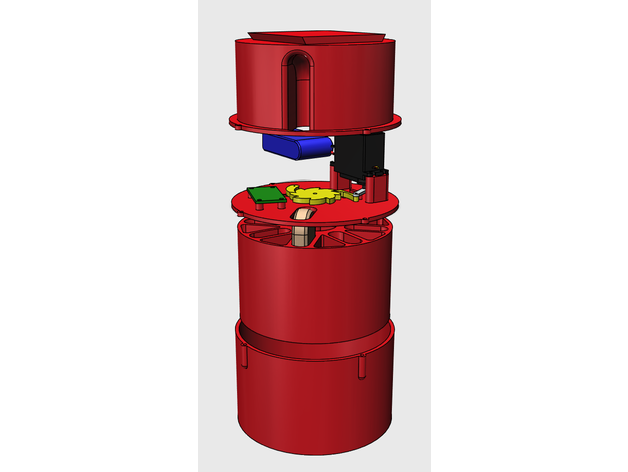

Supplement: S2 File — (ZIP) [file pone.0235548.s002.zip › images/EPD.png]

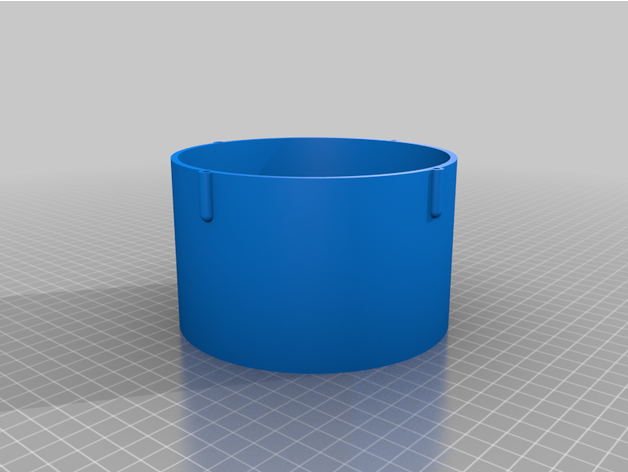

Supplement: S2 File — (ZIP) [file pone.0235548.s002.zip › images/XRT_base.png]

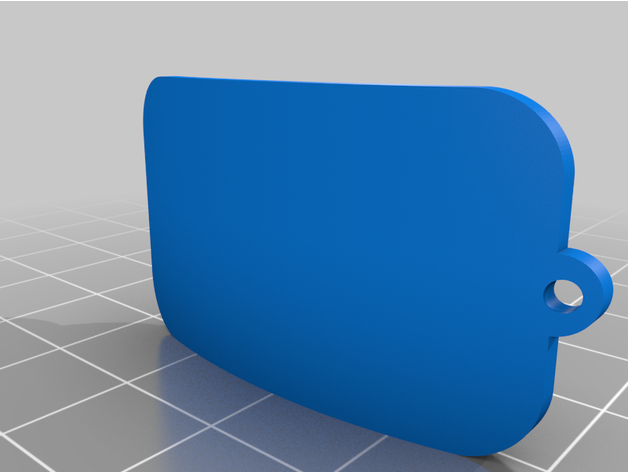

Supplement: S2 File — (ZIP) [file pone.0235548.s002.zip › images/XRT_batt.png]

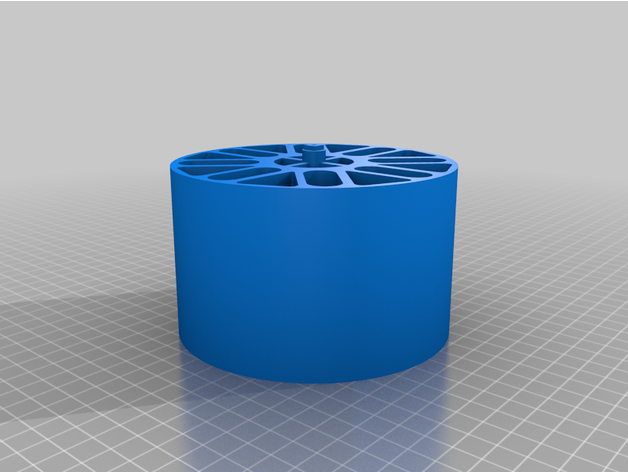

Supplement: S2 File — (ZIP) [file pone.0235548.s002.zip › images/XRT_carousel.png]

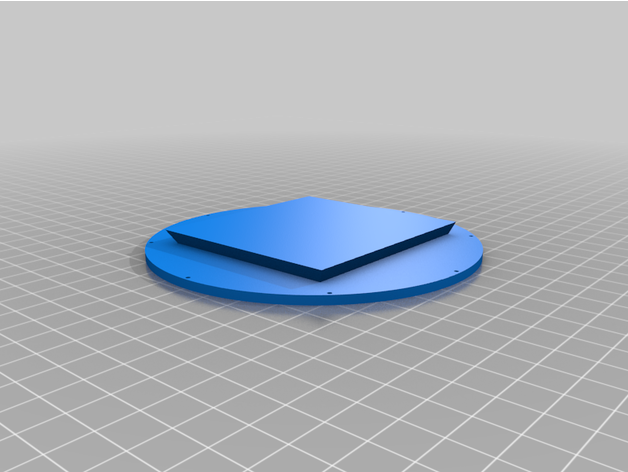

Supplement: S2 File — (ZIP) [file pone.0235548.s002.zip › images/XRT_cover-b.png]

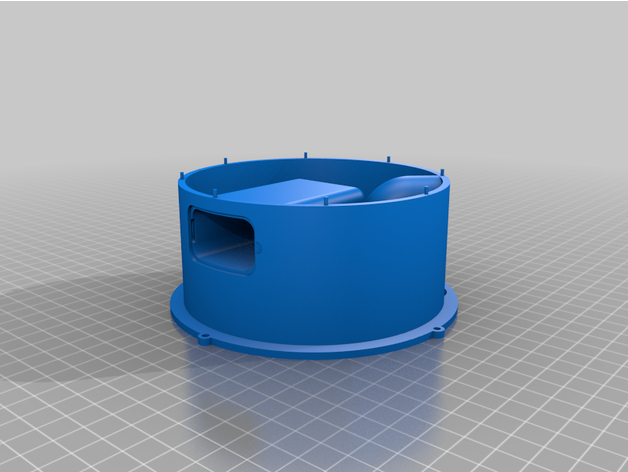

Supplement: S2 File — (ZIP) [file pone.0235548.s002.zip › images/XRT_cover.png]

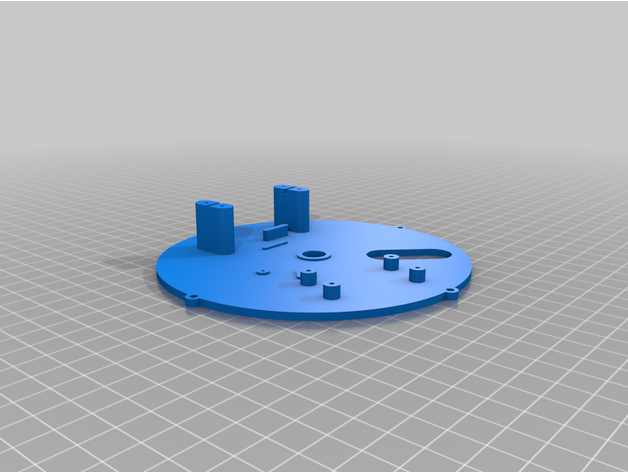

Supplement: S2 File — (ZIP) [file pone.0235548.s002.zip › images/XRT_lid.png]

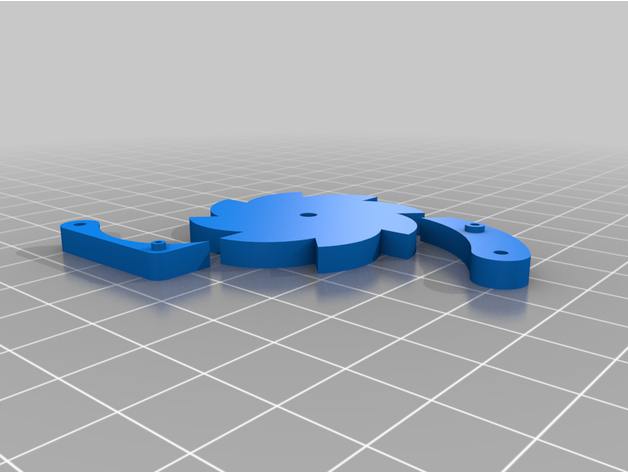

Supplement: S2 File — (ZIP) [file pone.0235548.s002.zip › images/XRT_ratchet.png]

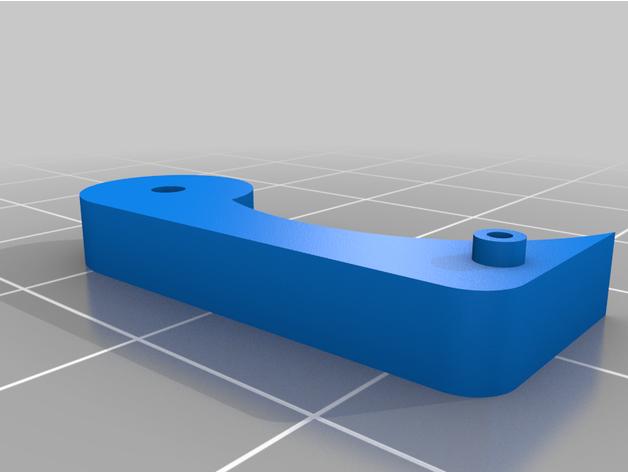

Supplement: S2 File — (ZIP) [file pone.0235548.s002.zip › images/XRT_ratchet_b.png]

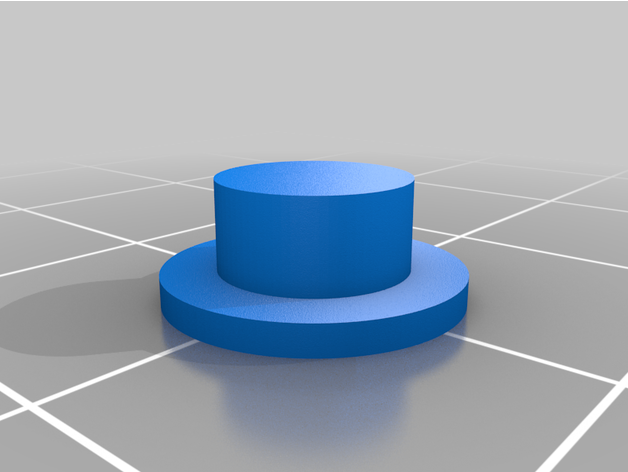

Supplement: S2 File — (ZIP) [file pone.0235548.s002.zip › images/plug.png]

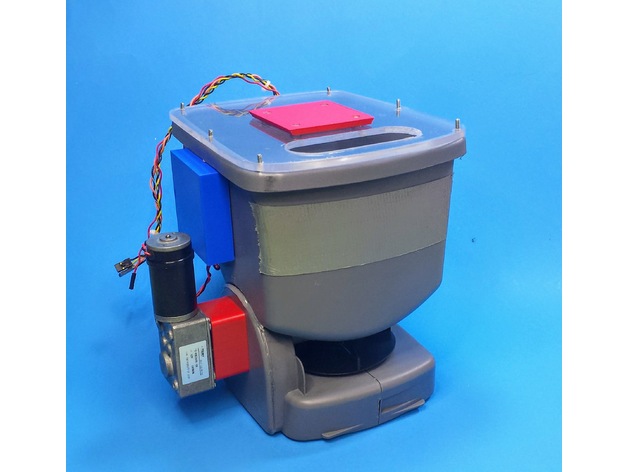

Supplement: S3 File — (ZIP) [file pone.0235548.s003.zip › images/20170130_182405.jpg]

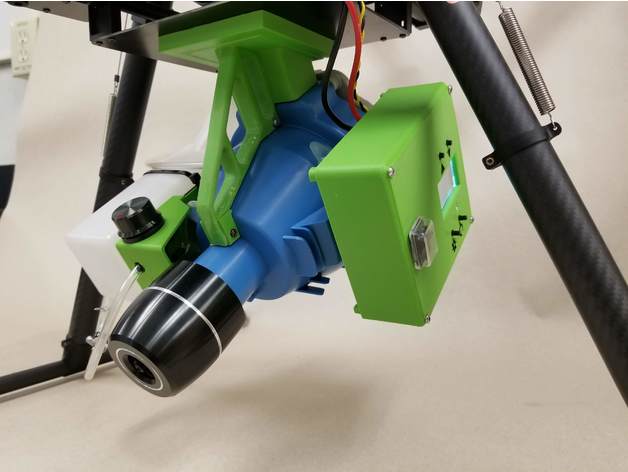

Supplement: S4 File — (ZIP) [file pone.0235548.s004.zip › images/20180907_120217.jpg]

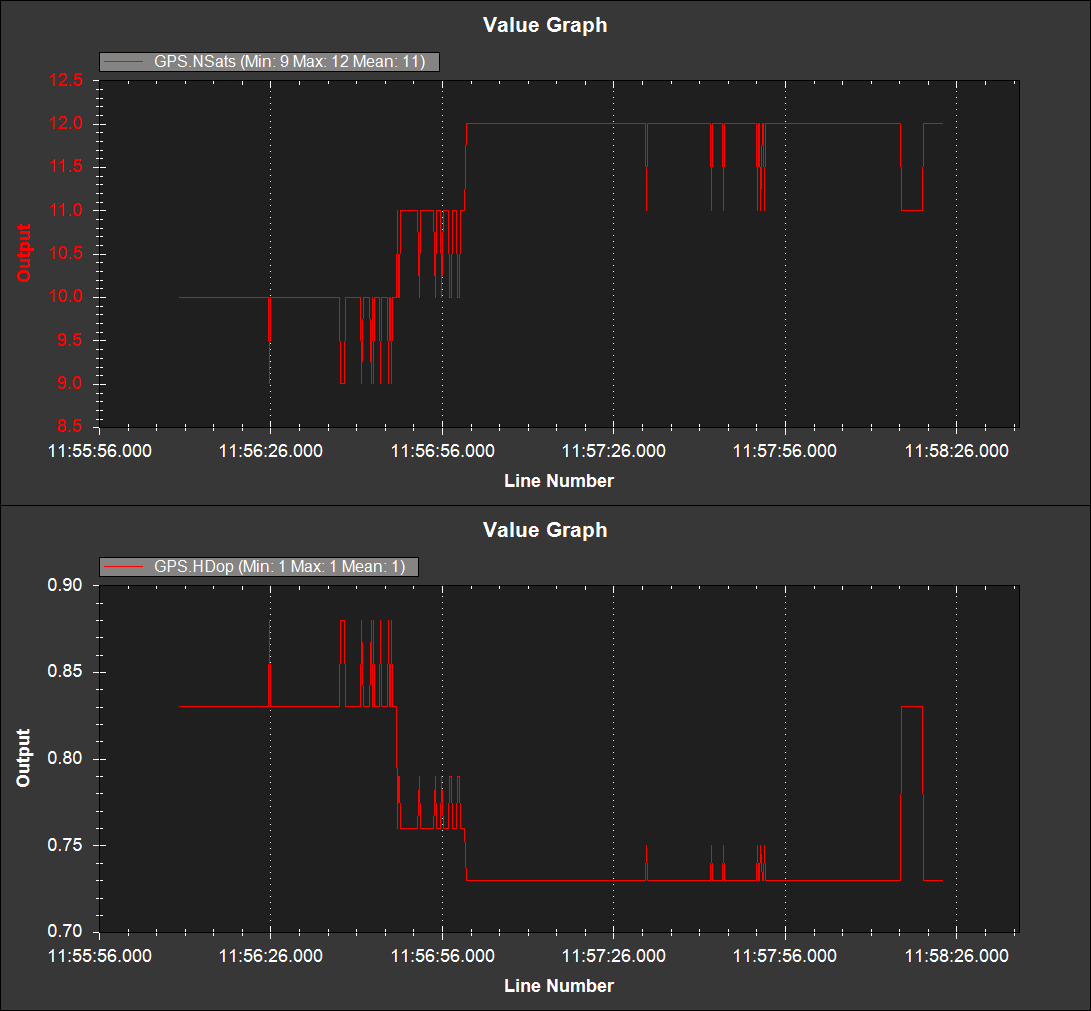

Supplement: S1 Fig — Total number of satellites broadcasting to GPS unit (NSats, top) and the horizontal dilution of precision (HDop, bottom). Lower HDop value indicates better GPS signal. Number of satellites increased and HDop decreased as flight time progressed. (TIF) [file pone.0235548.s005.tif]

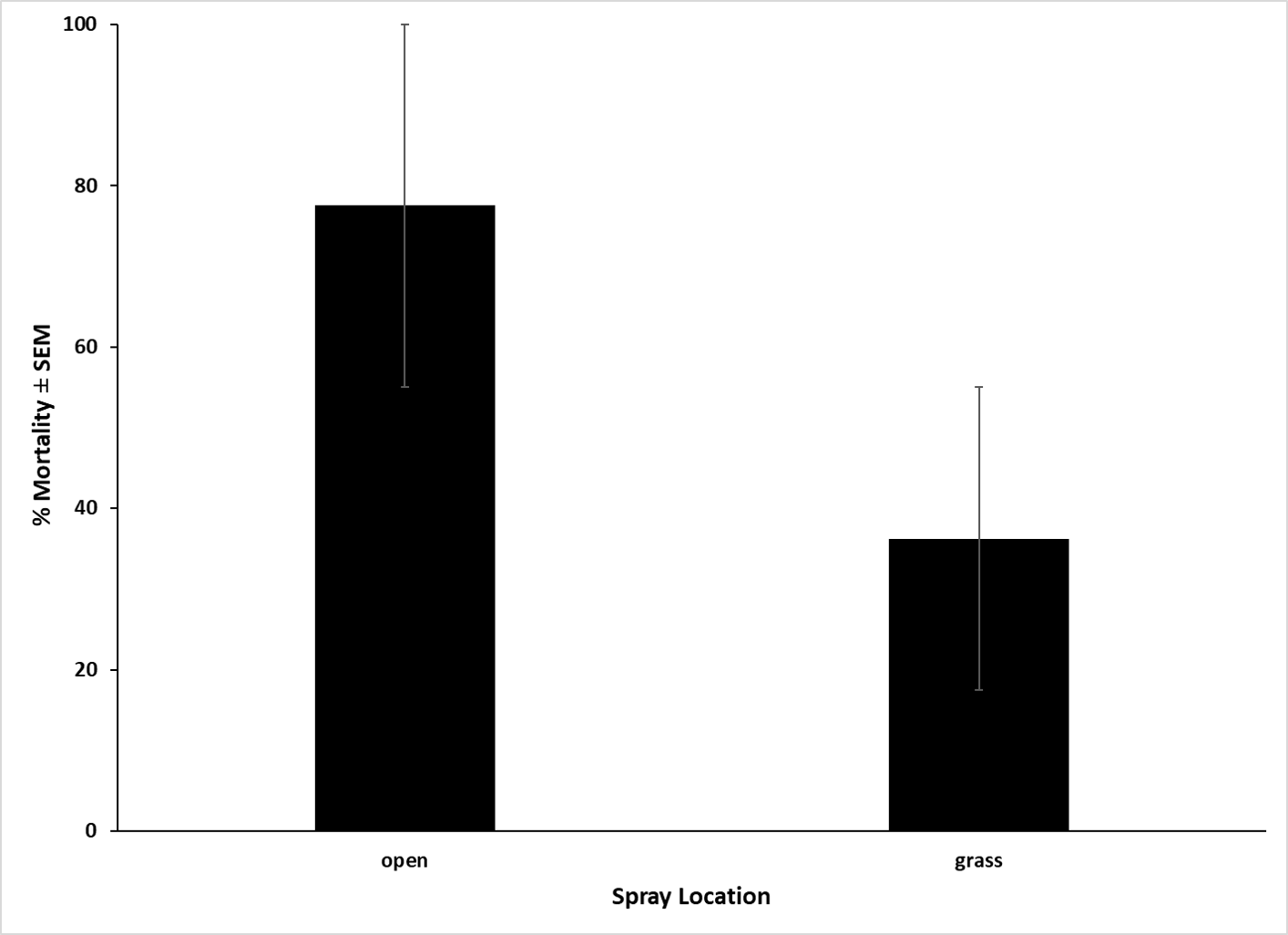

Supplement: S2 Fig — Average (n = 10) 72 hr larval mortality in cups placed out in open and cups placed beneath partial cover of Spartina patens grass. (TIF) [file pone.0235548.s006.tif]
