# Supplementary material for: The development of autonomous unmanned aircraft systems for mosquito control
Source: PLoS One. 2020 Sep 18;15(9):e0235548. doi: 10.1371/journal.pone.0235548 (PMC7500627; doi:10.1371/journal.pone.0235548)
Supplement: Attachment — Submitted filename: Response to Reviewers.docx [file pone.0235548.s008.docx]

**RE:** Ms. PONE-D-20-02700 “The development of autonomous unmanned aircraft systems…”

Dear Editor,

We have concluded a significant revision to the Williams et al. manuscript. We are greatly appreciative to the reviewers for their thorough critique of the work and believe the manuscript is much improved as a result. Our response to reviewer comments and suggestions are detailed below in red. I am confident that the revised manuscript meets the high standards of PLOS ONE.

Sincerely,

Greg Williams

Reviewers' comments:

**Comments to the Author**

1. Is the manuscript technically sound, and do the data support the conclusions?

The manuscript must describe a technically sound piece of scientific research with data that supports the conclusions. Experiments must have been conducted rigorously, with appropriate controls, replication, and sample sizes. The conclusions must be drawn appropriately based on the data presented.

Reviewer #1: Partly

Reviewer #2: Yes

2. Has the statistical analysis been performed appropriately and rigorously?

Reviewer #1: I Don't Know SAS files with statistical analyses have been uploaded.

Reviewer #2: Yes

3. Have the authors made all data underlying the findings in their manuscript fully available?

The [PLOS Data policy](http://www.plosone.org/static/policies.action#sharing) requires authors to make all data underlying the findings described in their manuscript fully available without restriction, with rare exception (please refer to the Data Availability Statement in the manuscript PDF file). The data should be provided as part of the manuscript or its supporting information, or deposited to a public repository. For example, in addition to summary statistics, the data points behind means, medians and variance measures should be available. If there are restrictions on publicly sharing data—e.g. participant privacy or use of data from a third party—those must be specified.

Reviewer #1: No Raw data, SAS files, and STL files have been uploaded.

Reviewer #2: Yes

4. Is the manuscript presented in an intelligible fashion and written in standard English?

PLOS ONE does not copyedit accepted manuscripts, so the language in submitted articles must be clear, correct, and unambiguous. Any typographical or grammatical errors should be corrected at revision, so please note any specific errors here.

Reviewer #1: Yes

Reviewer #2: Yes

5. Review Comments to the Author

Please use the space provided to explain your answers to the questions above. You may also include additional comments for the author, including concerns about dual publication, research ethics, or publication ethics. (Please upload your review as an attachment if it exceeds 20,000 characters)

Reviewer #1: Summary and overall impression.

The authors of “The development of autonomous unmanned aircraft systems for mosquito control” describe a custom-built UAS and pesticide application modules for mosquito control. Under PLOS ONE Criteria for Publication, this manuscript may be considered a submission that describes a tool (i.e. an UAS that applies insecticides for mosquito control). This manuscript is focused upon and describes in sufficient detail the deposition of pesticides by the UAS and pesticide application modules (e.g. swath width, pesticide deposition rates, etc). Therein, they describe the construction and benchmark testing of a relatively low cost UAS that is fitted with custom modules that apply liquid, granular, tablet and adulticide pesticides for mosquito control. The module that deposits tablet forms of pesticide is highly innovative as to the best of my knowledge, no such device is commercially available. This aspect alone may be of great use to the mosquito control community, particularly for using UAS to apply insecticide inside of containers which support the growth of mosquitoes that spread diseases such as dengue. However, a more rigorous evaluation of the automated tablet dispensing module would have benefited the study (accuracy assessed with a total of 12 tablets dropped from the UAS). The data presented for each module attached to the UAS were within ranges reported previously for other aircraft used for mosquito control. An important advantage of using UAS to apply insecticide is that it can provide a more targeted application, potentially reducing the quantity of pesticide released into the environment. Although the quantity of pesticide deposited is within the range that should control mosquitoes, the manuscript does not provide data on whether the test applications made using their custom UAS actually were effective for controlling mosquitoes. In line 516 of the manuscript, they indicate that mosquito control data will be presented in a future publication. However, it is of some immediate concern that vector control agencies may elect to follow the manuscript methods and construct a low-cost UAS for mosquito control, without adequate data to show that it is indeed an effective tool. If the described UAS is not effective, others attempting to replicate it will waste public funds, and potentially put the public at increased risk for mosquito-borne diseases. This concern is highlighted by the authors in lines 394-396 where they state that applications of the liquid larvicide VectoBac 12AS from their UAS produced inconsistent larval mortality. While theoretical comparisons were made between the custom UAS and other aircraft used for mosquito control, data was not presented that demonstrate the custom UAS has a proven advantage over existing alternatives.

Major Issues

- Much of the manuscript is dedicated to describing how to build the UAS with pesticide application modules and a specification section describing the results of evaluations on pesticide deposition rates from the UAS. The study unfortunately lacks information on the effectiveness of the UAS for controlling mosquitoes, except in line 394-396 where they state that the larvicide that was applied from the UAS produced inconsistent larval mortality. To diminish concerns regarding the efficacy of this instrument, larval cup bioassays and caged sentinel adult mosquito bioassay should be conducted for applications from each of the pesticide application modules. Testing the UAS configured with most effective parameters (speed, swath width, etc.) in a homogenous setting (e.g. open field) with one insecticide for each module should not be laborious.

We agree that bioassays would add valuable data. We have conducted bioassays for many of the UAS systems but have decided not to include those data. The overall length of this manuscript already limits the number of journals we can submit too. We feel that an additional eight sections in the Materials and Methods and Results and Discussions, with associated figures, would complicate an already long and technical manuscript.

In addition, as stated by Reviewer 1 below, “environmental factors can strongly impact the efficacy of pesticide deposited from an aircraft”. Therefore, any bioassays are as much a measure of environmental effects and pesticide efficacy as they are a test of the UAS systems. The application rates of pesticides are well-tested by the manufacturers, reviewed by the EPA, and are conservatively selected to deliver a lethal dose. As such, the use of blanks is common practice to evaluate equipment, independent of environmental or product variables. Several references are listed below highlighting the use of blank material for equipment characterization. Finally, as stated in line 472 of the manuscript, generalized application data allows for the extrapolation of the data to a wide variety of pesticides, not just the one or two tested.

[Hoffman et al. 2012](https://doi.org/10.2987/11-6109.1), [Farooq et al. 2016](http://www.dl.begellhouse.com/journals/6a7c7e10642258cc,41a95951399418ba,45a9694e70e0e7e3.html), [Breidenbaugh at al. 2009](https://www.researchgate.net/profile/Eric_Schoomaker/publication/277768401_The_United_States_Army_Medical_Department_Journal_July-September_2009/links/574efb0708aee47cb6e5350e.pdf#page=69), [Giles and Billing 2014](https://www.cabdirect.org/cabdirect/abstract/20143073873).

- The authors state in the submission questions that all relevant data are in the manuscript and Supporting Information files, but they do not provide the STL files that must have been used to program the 3D printer to make the pesticide application modules. Also not described is the software used to make the 3D models, or the 3D printer(s) that were used. These would be needed if other researchers attempt to replicate or build upon this study.

We apologize for the oversight. The STL files have been provided in the supplemental materials and the 3D printer and software have been referenced in the text, lines 262-263.

- The performance of the UAS and pesticide application system is compared to published articles and product manuals for other UAS used for pest control. Because environmental factors (e.g. wind, terrain, humidity) can strongly impact the efficacy of pesticide deposited from an aircraft, it would have been more compelling to compare the performance and efficacy of the UAS described in the manuscript with one that is commercially available and currently used in several industries (e.g. DJI Agras MG-1). A limitation of such comparison is that the Agras MG-1 has pesticide application modules for only liquid and granular products. However, application of tablets and adulticide by UAS is novel, so a direct comparison for these with another UAS may not be warranted.

We agree that a direct comparison to the DJI Agras MG-1 or other commercial systems would be useful. However, as stated, only the liquid spray systems could be compared. One purpose of this manuscript was to present a low-cost highly adaptable solution. As the adoption of UAS in mosquito control increases and more commercial options become available, direct comparisons will be more appropriate. We look forward to conducting those studies.

- The Discussion near the end of the manuscript is overly lengthy and not as focused as it is earlier in the manuscript. The end of the manuscript (lines 475-563) have been reduced in length and more sharply focused by removing unnecessary sections and rewriting several wordy sentences. While some of the writing may be speculative, we feel it is appropriate to alert the readers to the future potential of UAS.

Minor Issues

- Lines 37-38. The statement that the UAS applications were similar to applications from conventional aircraft could lead readers to think that actual comparisons rather than calculations that were made. This should be clarified. This statement has been revised to eliminate any confusion. “*UAS pesticide applications were made at rates prescribed for conventional aircraft, limited only by the payload capacity and flight time.”*

- Line 40: There is no data presented that pesticide use would be reduced with UAS applications, it is only inferred. Studies would need to be conducted to make this claim. The statement has been rewritten to reflect the inference. *“Despite those limitations, this system can deliver pesticides with much greater precision than conventional aircraft, potentially reducing pesticide use.”*

- Figures 1 – 3 do not present data, so could be included as Supplementary Information. This is a valid point; however we feel that the pictures are critical to understanding the described equipment and should remain in the main body of the text. We leave it to the discretion of the editor.

- Line 129: This reviewer searched the Cole-Palmer web site for “12V miniature gear pump” and received 44 results. Please provide the correct product number. Model number added. *“The liquid is pumped with a 12V miniature gear pump (EW-07012-20, Cole-Parmer®, Vernon Hills, IL)…”*

- Line 148-149: consider instead: “The liquid was collected and the volume that was dispensed measured using XXXX”; XXX being the instrument used to measure the volume. Statement updated as suggested. *“The liquid was collected and the volume that was dispensed was measured using a precision balance.”*

- Line 272-73; 309: Dv0.1, Dv0.5 and Dv0.9 are not described in the methods. Defined in methods, 163-165. *“Droplet spectra are described as the Sauter mean diameter, D_V0.1_, D_V0.5_ and D_V0.9_, which define the proportion of spray volume (10%, 50% and 90%, respectively) contained in droplets of the specified size or less.”*

- Line 279-80 The application rate of 0.09 L / ha is not suitable for Altosid SR-20 which the authors indicate is 0.07 L / ha. Curious why the authors would suggest the use of a product that could not be applied at the legal rate using the UAS. The application rate of 0.09 L/ha was a relative rate recorded on the spray cards. Based on our dozens of trials and discussions with Dropvision Ag representatives, it is not indicative of true application rates and should only be used as a relative measure of coverage. Those data have been removed from the figures and the section has been rewritten based on the true application rate of 1.6 L/ha.

- Line 304: Please indicate which system produced 50 um and which 123 um. Clarification added. *“…50 µm versus 123 µm for the Huang et al. (2009) system and our flat fan nozzles, respectively.”*

- Line 319: The authors claim that the system “is able to produce excellent efficacy” without direct evidence is not fully supportive of that statement. Sentence was rewritten to indicate that we expect excellent efficacy based on our data. *“…which indicate that our system can distribute larvicides at the proper application rate for common mosquito larvicides and should result in excellent efficacy.”*

- The application rate of 0.09 L / ha appears to exceed the maximum application rate for aerial release of Altosid SR-20 (according to the product label). This statement has been eliminated and the section has been rewritten as described for lines 279-280.

- Lines 320 – 329: Authors should make it clear they are speculating that 6 ha could be treated with a single battery charge based on calculations. This entire paragraph has been simplified and rewritten.

- 330: Authors may wish to refrain from grandiose statements such as “the days of blanketing an area with pesticide are numbered.” It is overly broad (area is not defined) and unlikely to be the case in the near to distant future as large areas such as marsh habitats, rice fields, or pastures are still likely to be broadly treated for mosquito larvae. Sentence removed.

- Line 332: Please describe or reference the field measurements that were performed to assess the distribution of *Aedes sollicitans* so that the statement here can be supported with data. Sentence was updated to reflect that measurements were based on manual dip counts of larvae. “*Based on our repeated manual dip counts of larvae in Spartina patens marshes,…”*

- Line 337: For Figure 8, only 12 tablet drops were reported. This is an unacceptably low sample size for a study designed measure the accuracy of an instrument, particularly since the study is simple to conduct (one tablet drop constitutes nearly 8 % of the total sample size). Unfortunately, with the current pandemic, all our flight fields are closed, and we are unable to assemble our research team for further experiments. We have updated the reported results with a 95% confidence interval and give a very conservative estimate (2.9 m) of the accuracy of the unit based on the farthest distance between any drops.

- Line 339: A description of ES could not be located in the manuscript. “ES” was referring to extreme spread but has been changed to “range” in the manuscript.

- Line 341 – 342: Authors state that wind had no effect on accuracy of tablet drop. However, one quarter of drop area did not contain tablets (270 – 360 degrees). This should be taken into consideration. Authors should indicate the wind direction on the figure to support that conclusion. Wind direction has been indicated in Figure 8 and the text has been updated to support the statement. *“…there was no apparent relationship between wind direction and tablet offset with most tablets landing northeast of the target despite a northerly wind.”*

- Line 345: R-squared values are reported, but the statistical software used to calculate it is not described in the manuscript. SAS software cited in M&M (lines 260-262) and listed in references.

- Line 353 – 355: The conclusion that the accuracy of the tablet dropper improved as flights progressed because the UAS had more time to collect and correct GPS data is not supported by data in the manuscript. The statement was revised to “*Accuracy improved as flights progressed because the UAS had more time to collect and correct for GPS data as indicated by the number of GPS satellites available and signal strength data recoded by the flight controller*” and a supporting figure was added to the supplemental materials.

- Line 386 – 387: The conclusion that releasing granules from 9 m was negatively impacted wind is not supported by the data as wind speed was not reported for this study and a reference indicating the wind speed that negatively affects granular drift is not provided. We removed our presumption of the effect of the wind from the statement. “*At 3 m altitude the swath was too narrow and at 9 m the larger, heavier VectoBac larvicidal granules had a swath of only 2 m.”*

- Line 400-404: The authors may want to also indicate the maximum application rate as vector control workers often use the maximum rate to reduce the potential for insecticide resistance to develop in mosquitoes. The maximum application rate has been added to the caption.

- Figure 11: This figure does not add a great deal to the study, and should be considered for removal. If the authors elect to retain this figure, they should describe how the predicted flight coverage (i.e. overlap analysis) was modeled. Figure 11 has been eliminated. The same information has been included in Figure 10 for clarity and the text has been updated accordingly.

- Line 416 – 417: The authors should provide data to support the claim that a newly designed granular spreader can treat 1.1 ha or remove the statement. We have clarified the sentence to explain that a 42% increase in hopper size will result in a 42% increase in area treated. “…*custom granular spreader with a 42% larger hopper, expanding the maximum area treated per flight from 0.76 to 1.1 ha...”*

- Lines 427-430: Indicating “unpublished data” appears to not be permitted by PLOS ONE. A supporting graph (S2 Fig) was added to Supplementary Information illustrating decreased larval mortality in bioassay cups placed beneath marsh grass.

- Line 433 – 434: Please provide the formula for calculating the estimated flow rate for VectoBac G in the RMAX. The statement has been amended using values provided in Miller 2005. *“…the flow rates were too high even at the lowest settings of the spreader, dispensing 16 kg of material in 20-30 min (533-800 g/min) (Miller 2005).”*

- Line 436 – 437: Speculating on the manufacturing plans for pesticide companies may be beyond the scope of this manuscript. However, using the data from this study to encourage the manufacturers to prepare formulations for use in UAS would be well within the scope. Sentence changed accordingly. “*We encourage pesticide manufacturers to develop lighter granules with a higher concentration of active ingredient specifically for UAS.”*

- Line 505 – 506: The statement that swarm technology will render payload capacity of UAS irrelevant is not supported by the data. Swam technology is likely to improve pesticide applications, but payload will likely remain an issue that will limit the duration of any application mission. We were referring to the combined payload capacity of the swarm. The sentence has been clarified. “*The combined payload capacity afforded through swarm technology will soon render the limited payload of single UAS irrelevant.”*

- Line 511 - 512: Please provide a reference supporting the statement that automated pesticide filling stations will be developed. If a reference is not available, it would be appropriate to encourage such development. (Stöcklin and Rauch 2017) reference added.

- Line 534 – 546: This section is overly lengthy and much is outside the scope of the study. We feel the legal issues surrounding the use of UAS are important, especially since many mosquito programs list regulatory issues as one obstacle to incorporating UAS into their programs. But given the likely changes to these regulations and in consideration of the length of the manuscript, this section has been deleted.

- Line 551: The reference Kimball & Reynolds 2016 is from a trade magazine that is not peer reviewed. There are peer-reviewed articles from the prior one or two years that may be more appropriate of a reference. Reference replaced with (Passos 2019).

- Line 552: Please provide a reference for this statement. Sample videos added to supplemental material.

- Several figures do not indicate whether the error is SEM or SD. All figures with error bars indicate SEM in the captions.

- A description of the statistics used in the study is not provided in the manuscript. Statistics are described in lines 260-265.

Reviewer #2: The paper entitled “The development of autonomous unmanned aircraft systems for mosquito control” by Williams et al. examines the delivery of insecticides for the control of larval and adult mosquitoes using modules developed for a UAS (drone) system. The paper provides a succinct history of unmanned aircraft use for mosquito control, discusses four modifications mostly made by adapting existing technologies with 3D printing technology, and provides data related to the distribution and (where relevant) droplet size for products applied using different altitudes, flying speeds and other parameters during the operation of the UAS.

The paper is well-written and provides an adequate quantitative summary and analysis of the results. I could not find any glaring shortcomings in the paper. The eleven figures seem appropriate. The references cited include many references that could be categorized as “gray literature” but they seem relevant to the text. The benefits and drawbacks of UAS technology relative to existing technologies are discussed and are summarized nicely. Given the specifics of the application results and mosquito control materials that are discussed, the paper might be more appropriate for a journal that focuses on mosquito control. Nevertheless, related papers have been published in PLoS ONE. I found this one to be an enjoyable read.
Minor criticisms are mentioned here:

Line 80: “…preferable to…” Corrected.

Line 117: “Data are…” Corrected.

Line 144: “alleged” flow rates? The connotation of alleged is “without proof, to have taken place or to have a specified illegal or undesirable quality.” The authors presumably do not mean the latter. Is the meaning of this statement to be that the flow rates were not confirmed or ground-truthed? We were referring to the flow rates advertised in the product literature. This sentence has been clarified. *“…flow rates specified by the manufacturer range from 0.32 – 4.83 l/min…”*

Line 170: “…servomechansim…” This should be servomechanism? Corrected.

Line 315: Coquillett Corrected.

Line 574: “…microhabitats…” Corrected.

Line 652: Is first letter of the surname of the first author an “I” or an “L”? (See also line 86). Good question, lol! It is an “I” as confirmed on the author’s ORCID ID. References have been updated.

Line 659: Correct format of the citation. Corrected.
