# Supplementary material for: The development of autonomous unmanned aircraft systems for mosquito control
Source: PLoS One. 2020 Sep 18;15(9):e0235548. doi: 10.1371/journal.pone.0235548 (PMC7500627; doi:10.1371/journal.pone.0235548)
Supplement: Attachment — Submitted filename: Response to Reviewers.docx [file pone.0235548.s009.docx]

**RE:** Ms. PONE-D-20-02700 “The development of autonomous unmanned aircraft systems…”

Dear Editor,

We have concluded a second revision to the Williams et al. manuscript. We have gone to great lengths to improve the manuscript and have reduced it by over 1,200 words. We find the reviewers’ comments fair and relevant and we are indebted for their detailed evaluation of this work. Our response to reviewer comments and suggestions are detailed below in red. We hope that the revised manuscript meets the high standards of PLOS ONE.

Sincerely,

Reviewers' comments:

Reviewer's Responses to Questions

**Comments to the Author**

1. If the authors have adequately addressed your comments raised in a previous round of review and you feel that this manuscript is now acceptable for publication, you may indicate that here to bypass the “Comments to the Author” section, enter your conflict of interest statement in the “Confidential to Editor” section, and submit your "Accept" recommendation.

Reviewer #1: (No Response)

2. Is the manuscript technically sound, and do the data support the conclusions?

The manuscript must describe a technically sound piece of scientific research with data that supports the conclusions. Experiments must have been conducted rigorously, with appropriate controls, replication, and sample sizes. The conclusions must be drawn appropriately based on the data presented.

Reviewer #1: Yes

3. Has the statistical analysis been performed appropriately and rigorously?

Reviewer #1: Yes

4. Have the authors made all data underlying the findings in their manuscript fully available?

The [PLOS Data policy](http://www.plosone.org/static/policies.action#sharing) requires authors to make all data underlying the findings described in their manuscript fully available without restriction, with rare exception (please refer to the Data Availability Statement in the manuscript PDF file). The data should be provided as part of the manuscript or its supporting information, or deposited to a public repository. For example, in addition to summary statistics, the data points behind means, medians and variance measures should be available. If there are restrictions on publicly sharing data—e.g. participant privacy or use of data from a third party—those must be specified.

Reviewer #1: No

The raw data (CSV files) and SAS output files have been uploaded with the revision.

5. Is the manuscript presented in an intelligible fashion and written in standard English?

PLOS ONE does not copyedit accepted manuscripts, so the language in submitted articles must be clear, correct, and unambiguous. Any typographical or grammatical errors should be corrected at revision, so please note any specific errors here.

Reviewer #1: No

We hope this is an error as the response to this question in the initial review was “Yes”. If not, we expect that the specific errors are outlined below.

6. Review Comments to the Author

Please use the space provided to explain your answers to the questions above. You may also include additional comments for the author, including concerns about dual publication, research ethics, or publication ethics. (Please upload your review as an attachment if it exceeds 20,000 characters)

Reviewer #1: This reviewer remains convinced that publishing this work without bioassay data that the authors apparently possess substantially limits the impact of the manuscript. Authors state that including bioassay data would complicate the manuscript. However, the authors provide a very lengthy discussion of data that is relatively simple to interpret and frame with the published literature (four figures with data: Fig 8 and 9: swath width / application rate; Fig 7: accuracy of dropping a briquette; Fig 5: liquid flow rate; Fig 6: droplet size / density).

Splitting the aeronautical / product deposition performance of the UAS and the bioassay data will require readers, if both are published, to flip between two publications to appreciate the value this research brings to the field. This reviewer sees that not combining the two as a missed opportunity to tell a complete story. If standard bioassay methods were used, they can be simply referenced in the methods to reduce the length and briefly compared in the manuscript with what is known for standard insecticide application equipment (via aircraft and truck).

However, if the authors remain steadfast on excluding bioassay data from this manuscript, this reviewer will respect that decision. I have provided comment below on how the manuscript must be revised to be acceptable for publication. A second revision is required because the Methods section is written in the present tense (past tense is standard) and the manuscript is overall very long considering the quantity of data presented. This affords the authors an opportunity to revise and condense other sections as well.

We maintain that bioassays are more a test of the pesticides and conditions, rather than the equipment. The choice of pesticides used, and species tested against would be arbitrary and incomplete. We thank the reviewer for their insight and appreciate their willingness to respect our decision.

The Methods have been rewritten in the past tense.

Major Issues

Over half of the abstract is introductory. Please condense. We have removed the introductory material and reduced the abstract from 283 to 174 words.

Introduction is somewhat long and introduces equipment or approaches that are not considered further in the manuscript. The Introduction should be condensed. We understand the reviewer’s concerns. However, the history of UAS in mosquito control is poorly understood and lies fragmented in several obscure journals. Our Introduction neatly summarizes this history, illustrates the progression of the technology, and explains the motivation behind this project. We stand behind the Introduction as written.

The Methods includes product specifications that can be easily obtained from the manufacturer and do not need to be republished in a research article. This includes lines 100-105; sentence starting line on line 107; 117-122; 143-145. The product specifications have been removed.

The discussion of the data is very long and must be better focused and condensed. For example, over 900 words are used to discuss differences in liquid flow rate through the nozzles, swath width, and droplet density + diameter (Fig 5 and 6). These are fairly simple measurements to discuss, and the literature (academic, manufacturer, and regulatory) is fairly clear on what is effective for making an insecticide application. This is the case throughout the manuscript, so authors must examine the manuscript in its entirety to condense and anchor the writing in the data they collected. The Results and Discussion section has been reduced from 2950 to 2350 words. In particular, the liquid sprayer section has been reduced from 847 to 628 words.

Overly lengthy discussion of data muddies the message. Authors should determine key messages they wish to discuss, and discard what is tangential or not crucial and supportive. Please see response directly above and below for Conclusion section.

Fig 1, 2, 3, and 4 should be combined into a single multi-panel figure (e.g. Fig 1A, B, C, D). Figures 1-4 have been combined into a single figure and the caption has been updated accordingly.

Figure 5 and 6: These figures are related and should be combined into a single figure with panels A and B. Figures 5 and 6 have been combined into Figure 2 and the caption has been updated accordingly. Labels for other figures have also been updated in captions and text.

Fig S3 , S4: These are links to YouTube videos of drones imaging mosquito larvae under water. They are not relevant to the results presented in the manuscript or derived from a peer-reviewed scientific study. Additionally, it is impossible for viewers of the video to know anything of the equipment, and thus are of no value for comparison to anything in the manuscript. This supplementary information must be removed from the manuscript. This section has been eliminated.

Lines 261 – 263: Authors state the software used to program the 3D printer, but do not appear to have provided the STL files. Their response to this reviewer state “The STL files have been provided in the supplemental materials”, but they were not in the Appendix of supplementary figures (lines 647 – 655) or mentioned in the Materials section. As stated previously, these files will be needed by other researchers if they wish to attempt replicating what is presented in this manuscript. The model files must be provided by the authors and they must state in the Methods section where they can be downloaded. We apologize for the confusion. We intended to upload the files to Plos One with the submission, but the STL files are nearly useless without the software to view them properly. All STL files have been deposited in Thingiverse.com under file numbers 4427999, 4428029, 4428081, and 4428111 and are available free for download. Links have been added to the supplemental materials.

Line 456 to end of manuscript: This section of the manuscript should start with a “Conclusion” subheading as it is not restricted to discussing the ULV module. It is also very long (nearly 1,000 words), especially considering there are four figures with data. This section will benefit a great deal by condensing the writing substantially and focusing on what can be concluded overall from the studies. Much of what is provided in line 461-end is, for the most part, tangentially related to the results and discussion. The authors may wish to consider instead of revising this section, end the manuscript with line 460 as it would simplify and speed the revision process. It is of course up to the authors how to revise this section, but it must be condensed substantially. A “Conclusion” heading has been added. There were only two figures in this section, and both have been removed. The Conclusion section has been reduced from 987 to 737 words. We include this section because we feel it is appropriate to contrast UAS to full scale aircraft, especially considering this is the primary comparison many mosquito control professionals will make. And while tangentially related to the R&D section, our vision for the future of UAS in mosquito control is appropriate will hopefully shape subsequent research.

Minor Issues by Line Number

41: Speculative to state that UAS are preferred in congested environments as manned aircraft are very effective in such areas. Rephrase to “may be preferable”. Sentence updated to “In smaller, congested environments or in programs with limited resources, UAS may be a preferable alternative to conventional aircraft”.

105: Irrelevant what controller is usually used with the UAS; the controller used in the study should be the focus. Sentence updated to “The UAS was equipped with a Pixhawk® (3D Robotics®, Inc., Berkeley, CA) flight control system (FCS) because it offers flexibility in programming and controlling peripheral devices”.

138: liters is typically abbreviated with “L” not “l”. Abbreviations have been updated accordingly.

149: state manufacturer, model of precision balance. Sentence updated with “(model PX523, Ohaus Corp., Parsippany, NJ)”.

187: delete and append to prior sentence: (n = 3 replicates) Done.

190: manufacturer and model of video goggles? Model information added “(model Dominator HD3, Fat Shark Vision Systems, Qinghai, China)”.

213-214: This is data and should be moved to Result section The sentence was rewritten as “The larger Altosid pellets were tested with the gate at 7, 10, and 20 mm open.”

215: see comment for line 187 Updated as above. Also done for lines 149 and 253.

226: delete “p” at end of line. Done.

231: State parts were removed. Impossible for others to replicate the work without knowing. If lengthy, it can be included as a supplementary table / figure. The sentence was updated to reflect that the housing and spray tank were removed.

233- 239: Very lengthy; please condense. This section has been reduced from 114 to 74 words.

275-278: Unclear if the authors made the measurements or if they are calculations. Values in parentheses are presumably label application rates; if so it should be stated. Sentence was updated to: “Based on our calculations, VectoBac WDG (label rate: 2.4 – 93.5 liters/ha) can also be applied…” Values in parentheses were updated to reflect that these are label rates.

297: Affect of changing nozzles on swath width was not measured. It is an overstatement that doing so “will”. “Will” has been replaced with “should”.

299: If this is in reference to data in a figure, the figure number should be stated. There is no figure associated with this data.

311: If this is not the result of an experiment, it should be indicated as speculative with “this system should cover”. Sentence was updated as requested.

314: Readers that are not experts in mosquito control will not understand “dip counts” as a measure of larval mosquito abundance. This needs to be explained in the methods. Averages of data that are presented in a manuscript must include error measurements. Such error values are absent here. This section has been deleted.

316-317: Describe how the reduction in pesticide use was calculated. This section has been deleted.

348-349: Data indicates that autonomous is more accurate and thus should be preferred. If authors interpret the data otherwise, this statement should be offered as an opinion and not a direct interpretation of the data. This section (and corresponding M&M) was removed to focus the manuscript on the autonomous capabilities of the UAS.

390-394: Authors are introducing results that are not supported by data in this manuscript. Please remove. Section removed.

498: Passos 2019 is a PhD thesis, which is unusual to include as a reference for a peer-reviewed journal article. After examining the thesis, the student analyzed imagery that was reported in a student design competition “IEEE CAS Student Design Competition 2017–2018 Finalist project”. While I’m sure there are examples of referencing PhD theses in manuscripts, there must be at least one peer-reviewed article in the literature that is more robust and relevant that can be referenced instead. Passos 2019 was replaced with Case et al 2020.

S2 Fig: Y-axis label is not standard. Please correct. The figure has been updated.
